# Development and Influencing Factors of International Trade in Digitally Deliverable Services

**DOI:** 10.3389/fpsyg.2022.908420

**Published:** 2022-05-24

**Authors:** Yuna Di, Ruixin Zhi, Huaixi Song, Lu Zhang

**Affiliations:** School of International Economics and Management, Beijing Technology and Business University, Beijing, China

**Keywords:** digitally-deliverable services, digital infrastructure, human capital, artificial intelligence, e-commerce, online consumer behavior, social commerce, international trade

## Abstract

**Introduction:**

International trade in digitally deliverable services has been developing rapidly in recent years, especially during pandemics. However, the growth rate and scale differ among regions and countries. To promote the growth and bridge the divide of global trade in digitally deliverable services, we provide feasible policy suggestions in this paper.

**Methods:**

Based on panel data of 33 countries' digitally deliverable service trade from 2005 to 2020, this study analyzes the development trends and influencing factors of international trade in digital-global service trade.

**Results:**

The development of digitally deliverable services in developed countries is far ahead, while in emerging economies such as China and India, it is growing at a high speed. Digital infrastructure; human capital; and science, technology, and innovation capacity have a significant impact on the digitally deliverable trade of countries with the level of STI playing the most important influence. Further, heterogeneity analysis shows that human capital and STI capacity are more sensitive to the digitally deliverable trade of middle-income countries than to the impact of high-income countries, and the contribution of population in middle-income countries is more pronounced.

**Conclusion:**

As the proportion of digital trade in global trade continues to increase, the role of digital service trade in countries' sustainable development is becoming more prominent. To cope with such opportunities and challenges, countries, especially developing countries, should actively increase digital infrastructure construction, improve talent training, vigorously develop digital technology, and enhance their innovation capacity.

## Introduction

In the context of profound changes in the speed and shape of economic development contributed by the technological and industrial revolution, particularly the advancement in digital information technology such as the Internet, big data, and artificial intelligence, the digital economy has come into being and gradually become the main component of the national economy of each country. Meanwhile, the unbalanced development of the digital economy among different countries will further aggravate the imbalance in world economic development, contributing to the emergence of the digital divide (James, 2007). As the digital economy is booming, data has become a basic and strategic factor of production in economic activities, the trade exchange of elements, contents, and services in the form of data has become more active, such as digital entertainment, cloud computing services, blockchain services, etc. (González and Jouanjean, 2017). Furthermore, data can itself be traded as an asset and generate a means through which global value chains are organized and services trade delivered, thus facilitates the co-ordination of global value chains and extends the international division of labor from the physical world to the digital world, connecting a great number of businesses and consumers globally. On the other hand, information and communications technology (ICT), which also profoundly reshapes the process and mode of trade, has disrupted traditional trade of various aspects. Digitalization has not only increased the scale and scope of trade, but has also diversified trade bodies, as evidenced by the increased number of small-scale production firms using ICT to overcome transaction challenges and provide products and services to digitally connected customers with specific preferences around the world. Besides, trade is no longer strictly restricted by geographical distance, time, and language, and the negotiation, ordering, and delivery procedures aspects of trade are conducted online, reducing trade costs while improving trade efficiency (Miroudot and Cadestin, 2017). While there is no single recognized and accepted definition of digital trade, it has universally been talked about. In order to better define and understand the subject of this paper, we make a distinction between the concepts that have been widely used, through the dimension of objects of trade (what) and how trade is conducted (how), so as to clarify the caliber of digital trade described in this paper: When the mode of trade is non-digital, no matter the object of trade is digital or non-digital, it can be classified as traditional trade; and when the mode of trade is digital and the object of trade is non-digital, according to the OECE and WTO definitions, it is closer to the category of e-commerce, which is considered to be part of the broad scope of digital trade; only when both the mode of trade and the object of trade are digital, according to the USITC definitions, it can be categorized as digitally deliverable services trade (DDS), which is considered to be narrow scope of digital trade. Furthermore, from the perspective of development stage and trade characteristics, scholars often consider digital trade as an advanced form of cross-border e-commerce development. In contrast to cross-border e-commerce, digital trade tends to pay more attention to consumer behavior and accumulates and applies big data on the consumer side from the perspective of big data by matching more digital tools and means, which can actively reflect consumer preferences (Ma et al., 2018).

**Figure 1:**
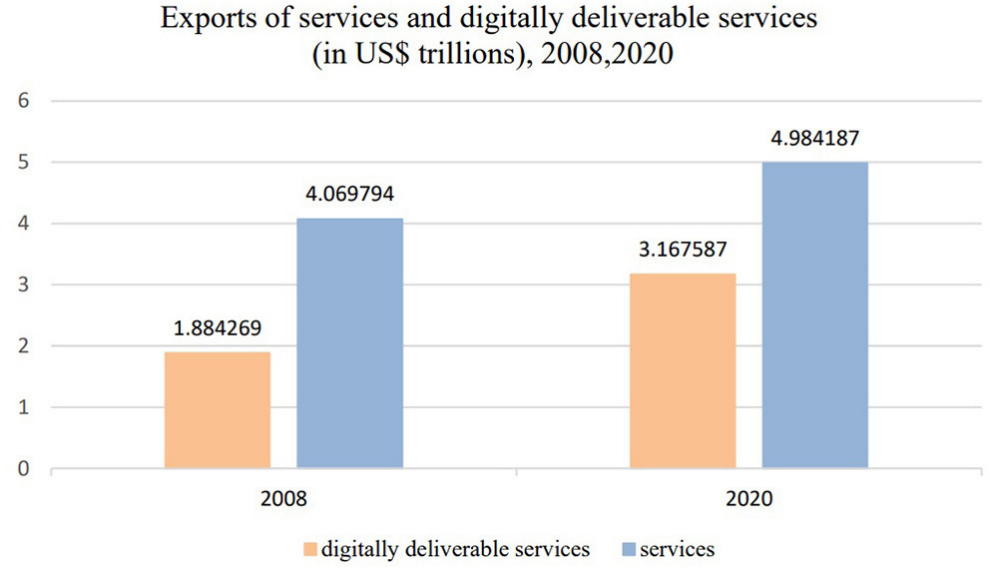
Exports of services and digitally deliverable services (in US$ trillions), 2008, 2020.

**Figure 2:**
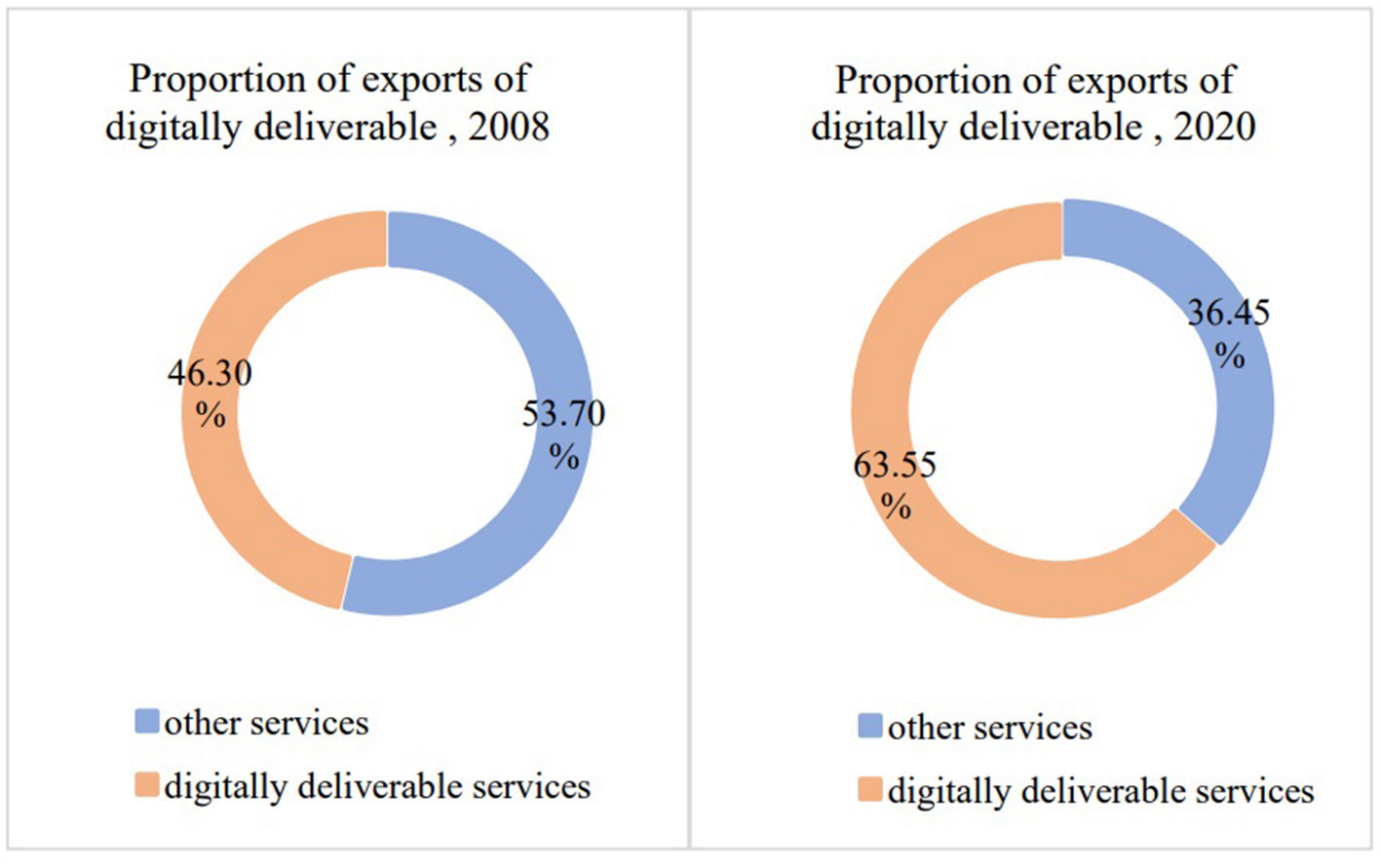
Proportion of exports of digitally deliverable services, 2008 and 2020.

As Fu (2020) points out, the coronavirus disease 2019 (COVID-19) pandemic has accelerated the digital revolution and sped up the process of global digital trade. In the immediate aftermath of the COVID-19 outbreak, governments worldwide issued lockdowns, quarantines, restrictions, and closures orders. In order to maintain the rhythm of life and studying, the public need to seek alternative methods including telemedicine, virtual classroom, online shopping, social interactions and working remotely which involves all aspects to conduct lives while in isolation. All of these require access to internet and digital technologies, thus digital trade especially the digitally deliverable services have rapidly increased. Compared to the figures of 2008 and 2020 sourced from the “International trade in digitally deliverable services (DDS)” released by UNCTAD, the international trade in digitally deliverable services exports grew from about US$1.88 trillion to around US$3.17 trillion (see Figure 1), and its share of global services exports grew from 46.30 to 63.55% (see Figure 2), indicating that digital trade has become a new major driver of global services trade growth.

**Figure 3:**
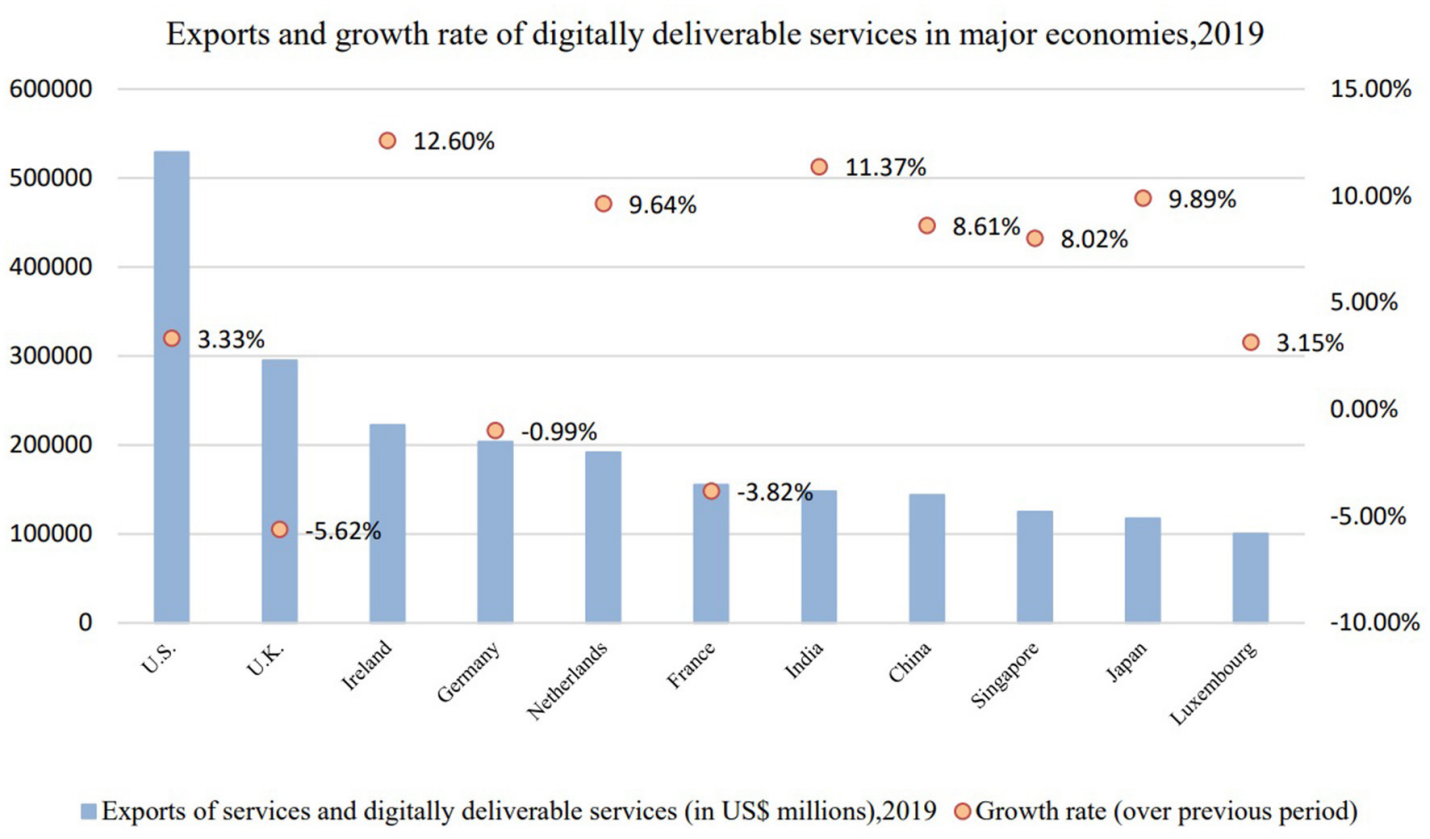
Exports and growth rate of digitally deliverable services in major economies, 2019.

Meanwhile, a report from UNCTAD (2021) reveals that the COVID-19 pandemic has also exposed the highly uneven global digital transformation among countries (as shown in Figure 3) to be specific, with countries such as the US, UK, and Ireland ranking at the top in deliverable digital services trade exports, while India, Japan, and China were among the top in terms of deliverable digital trade growth rate, standing at 11.37, 9.89, and 8.61%, respectively. It can be possibly aware that lower income countries were likely to face digital obstacles due to lacking of access to internet as well as inadequate knowledge and independent use of the digital devices. However, these countries enjoyed a high level of development potential.

With regard to the study of digital trade in the context of the digital economy, existing studies, which tend to conduct comparative analysis from the perspective of international differences, have put relatively less emphasis on the influencing factors of digital trade, especially lacking the analysis of influencing factors constructed through econometric models. It is of great importance to point out that this study aims to pinpoint the commonalities among the differences and identify the critical factors affecting digital trade by comparing digital trade differences among countries, based on which analysis has been carried out on how to optimize organization and management at the national and enterprise levels, with the aim of facilitating digital trade for countries that are temporarily lagging behind (Sharma and Gupta, 2003), bridging the digital divide at the digital trade level, and giving better play to the facilitating role of digital trade in the economy. At the same time, the study of digital trade can also provide opportunities for many small and medium-sized enterprises (SMEs) to participate in the international market through digital technology, supporting them in achieving better performance in a trade pattern featuring increasing instability and uncertainty, and further promoting healthy competition in the market.

This analysis unfolds in the following sequence: section Literature Review defines the scope and measurement of international trade in digitally deliverable services based on the existing literature and reports, and identifies the influencing factors that feature authority, data availability and global data comparability concerning digitally deliverable services. Section Data and Methodology details the variable selection and data sources as well as the econometric model. Section Results presents the regression estimation results, including a robustness and heterogeneity test analysis. Section Discussion discusses relevant studies and highlight this paper, followed by the conclusions as well as policy suggestions in section Conclusion.

## Literature Review

### Scope of International Trade in Digitally Deliverable Services

Since the concept of digital trade emerged, scholars have explored its connotations from different perspectives. As Weber (2010) mentions, digital trade refers to the transmission of valuable products or services through electronic delivery. As pointed out by Wang (2021), digital trade is a new form of trade that features digital technology as its means, digital services as its core, digital platforms as its support, and digital delivery as its main channel. Ma et al. (2018) focus on the discussion from the perspective of digital trade that has accelerated the industrial revolution and consider digital trade as a type of trade that applies modern information networks as its carrier to achieve the effective exchange of physical goods, digital products and services, and digital knowledge and information through the utilization of ICT, thus transforming from a consumer-oriented Internet to an industry-oriented one, and eventually achieving intelligent manufacturing.

In addition to the attention drawn from academia, digital trade has been frequently mentioned in the reports of international institutions, government departments, and business organizations, although there is no unified connotation of digital trade in the international arena. Broadly speaking, it is divided into two schools based on the scope of the subject matter of digital trade transactions. Among them, the narrow connotation of digital trade was first proposed in Digital Trade in the United States and the Global Economy (Part I) (International Trade Commission of the United States, 2013) released by the United States International Trade Commission (“USITC” for short) in 2013, and was later reaffirmed by its report entitled Global Digital Trade: Market Opportunities and Foreign Trade Restrictions in 2018 (International Trade Commission of the United States, 2018), pointing out that the digital trade refers to “trade in services delivered digitally,” and its subject matter mainly consists of intangible services, information, and data, excluding goods ordered online and physical goods with digital counterparts. On the basis of this, it also summarized the three characteristics of digital trade are as follows: First, its transaction is based on Internet technology; Second, the subject matter of trade is intangible knowledge and technology-intensive digital products or services; Third, the marginal production and transportation costs are almost zero.

In a broad sense, the concept of digital trade is mainly defined by international institutions led by the Organization for Economic Cooperation and Development (OCED) and the World Trade Organization (WTO), which consider digital trade as products and services ordered, produced, or delivered through the Internet and other digital technologies. This concept covers not only service products delivered digitally but also physical goods traded through information and communication technologies and digital means.

In this study, international trade in digitally deliverable services focused on intangible digital product services delivered through the Internet, excluding physical objects.

### Measurement of International Trade in Digitally Deliverable Services

The measurement of international trade in digitally deliverable services are presented by the United Nations Conference on Trade and Development (UNCTAD) and the US Department of Commerce (USDOC). On the basis of the classification of balance of payments services extended in the Balance of International Payments and International Investment Position Manual (6th edition) (IMF, 2013), UNCTAD and USDOC argue that the subject matter of transactions in digital trade consists of the services subheadings of the Balance of Payments (BOP) that can be delivered in digital means, including insurance and pension services, financial services, charges for the use of intellectual property, telecommunications, computer and information services, other business services, and audiovisual and related services. As for the accounting of trade in digitally deliverable services, the Grimm measure was proposed by the American scholar A N Grimm in 2016, according to which the trade in digitally deliverable services is divided into ICT services, potentially ICT-enabled services, and non-potentially ICT-enabled services.

The trade measurement of multidimensional architecture is mainly proposed by the OECD and WTO in the Digital Trade Measurement Manual (1st Edition) (OECD et al., 2020), which defines digitally ordered transactions, digitally delivered transactions, and digital intermediation platform-enabled transactions, among which digitally ordered services are the part that does not overlap with the narrow caliber; that is, it includes the part of goods that cannot be delivered digitally but can be ordered digitally, broadening the scope of the trade in digitally deliverable services under the narrow caliber.

This study adopts the definition of digitally deliverable services trade by UNCTAD and USDOC when measuring digital trade.

### Factors Influencing Trade in Digitally Deliverable Services

Considering that the concept of digitally deliverable service trade is newly proposed, the influencing factors of traditional service trade and the characteristics of the digital economy are combined in this analysis to propose factors that may affect digitally deliverable service trade and to put forward the corresponding measurement indicators.

#### Digital Infrastructure

The digital economy has broken down information barriers to a larger extent and reduced the geographical limitations of economic activities, creating a huge digital dividend through inclusion, efficiency, and innovation, all of which presuppose digital connectivity. The digital economy model stimulates demand for infrastructural reliability, which accelerates the reproduction process. Henfridsson and Bygstad (2013) pay attention to the generative mechanisms of digital infrastructure through an in-depth case study and a case survey, distinguish that “adoption, innovation, and scaling” are three key mechanisms of digital infrastructure evolution and then discuss the successful evolution way of digital infrastructures. The COVID-19 pandemic had seen a great effect on the societal resilience and business continuity owing to digital connectivity, Strusani and Houngbonon (2020) point out that digital infrastructure providers in emerging markets may face more drastic competition. Digital infrastructure provides fundamental support for the development of digital trade, and the level of a country's digital infrastructure often constrains the development of digital trade (Wang, 2021). Internet penetration and the number of people with access to the Internet also affect digital trade. As Freund and Weinhold (2002) point out, an increase in the share of U.S. trading partners with access to the Internet is conducive to U.S. service trade exports.

#### Human Capital

Digital industries feature highly intensiveness of knowledge. As Coleman (1988) states, human capital is created by changes in personnel that bring skills and competencies, enable people to act in new ways, and contribute to productive activities to some extent. Schultz (1961) proposed that investment in human capital can improve the quality of human effort and enhance productivity. As shown in a study on a panel of around 100 countries observed from 1965 to 1995 by Barro (2001), both the quantity and quality of education affect long-term economic growth, and they also suggest that human capital plays an important role in the diffusion of technology. Wößmann (2003) reviewed the measurement of human capital in empirical growth research, which includes factors such as adult literacy rates, school enrollment ratios, and average years of schooling of the working-age population and etc., and pointed out that different specifications lead to different measures of human capital across countries. Lutz et al. (2017) systematically and quantitatively addressed the role of educational attainment in global population trends and models, arguing that future educational attainment levels are key determinants of outcomes, ranging across economic growth, quality of governance, and adaptive capacity to environmental change. Reddy and Gairola (2002) targeted the Indian service sector and pointed out that the important factors affecting the international competitiveness of trade in services are the level of education and mobility of talents.

#### Science, Technology and Innovation (STI)

As digital trade is different from traditional trade in goods and services, digital technology is often the core driver of digital trade development. Digital industries are technology-intensive, and innovation is the driving force and guarantee of digital technology development (Rogers, 1995). As Guerrieri and Meliciani (2005) argues, the competitiveness of a country's trade in services is determined by the state of its manufacturing structure and is positively related to ICT development. As shown in Freund and Weinhold's (2002) study on US service trade, ICT development is beneficial to a country's digital service exports. The modern theory of sustainable economic growth is based on innovation (Nurpeisova et al., 2020). Further, as confirmed by Windrum and Tomlinson (1999), the competitiveness of the service trade and the degree of innovation are positively related. Based on data from developing countries, Forero-Pineda (2006) finds that stronger intellectual property rights protection has a negative effect on science and technology. Lee (2011) explored the relationship between trade and innovation using data from the Malaysian manufacturing sector covering 1997–2004 and found that both product and process innovation have a strong effect on exports.

#### Other Factors

First, the digital economy highlights the importance of market size owing to economies of scale and network effects (Deardorff, 2017; Knudsen et al., 2021). The larger the market size, the higher the benefits of marketing and model optimization for all types of enterprises and the greater the value of developing niche markets and long-tail markets. In addition, a large number of digital consumers and users on the demand side can generate a large volume of underlying data, which can support enterprises in forming core competitive advantages. As for this economic effect, this study adopted a population metric for measurement. Second, many countries have adopted different attitudes toward “cross-border data flow,” considering economic and data security, which has led to many controversies in the negotiation of international trade rules between countries. The degree of trade openness, especially in the field of data openness policy, has become an urgent factor to consider. In addition, Ahmedov (2020) focused on changing the structure and forms of international trade and pointed out that it is necessary for countries to expand international cooperation and actively participate in digital commerce in foreign markets. The degree of trade openness also affects the ease of market access and fair competition market order to a certain extent, which in turn affects the business environment of digital trade, and more open trade provides good conditions for the development of digital trade (Crenshaw and Robison, 2006). Considering this backdrop, this study adopts an indicator of trade openness to measure this factor. Third, we considered foreign direct investment. Porter (2009) argues that the presence of strong domestic competitors is a key stimulus for firms to achieve sustained competitive advantage in his Diamond Model, and that the adoption of different strategies in other countries on the same firm type as well as organizational structure can also have an impact on firms' competitive advantage. As demonstrated by Dash and Parida (2013), from the perspective of business strategy, the relationship between foreign capital inflows and services exports turns out to be complementary at the aggregate and sectoral levels. As Wang (2021) argues in terms of the development factors affecting digital trade in China, multinational companies control the core of the data value chain and therefore have a significant impact on digital trade. Based on this, this study adopted foreign direct investment indicator for measurement.

## Data and Methodology

### Data Collection and Processing

This study selects data on exports of digitally deliverable services trade of 33 countries from 2005 to 2020, including Australia, Austria, Belgium, Brazil, Canada, China, Denmark, Finland, France, Germany, Greece, Iceland, India, Indonesia, Ireland, Italy, Japan, Korea, Luxembourg, Mexico, Netherlands, New Zealand, Norway, Poland, Portugal, Russia, Singapore, South Africa, Spain, Sweden, Turkey, the United Kingdom, and the United States of America. First, these countries cover the world's major economies, consistently ranking among the top in the list of global economic volume, and covering the world's five continents: Asia, America, Africa, Europe, and Oceania. Second, according to the World Bank's classification of the world's major economies, the chosen countries cover 25 high-income countries and 8 middle-income countries, which adopt the combined criteria of gross national income per capita, geographical location, loan eligibility, and economic vulnerability; thus, it would be applicable in our subsequent work of group heterogeneity to test models. In addition, before obtaining the raw data and adding them to the model regressions, this study carried out logarithmic calculations of all data to avoid bias in the model regressions due to inconsistencies in the data magnitudes.

#### Dependent Variable

Nicholson and Noonan (2017) identified “digitally deliverable” services in digitally enabled service categories and presented an upper-bound estimate of the percentage of digitally deliverable service exports. Aliev et al. (2020) examine new digitalization trends in the context of international trade development and emphasize the importance of increasing the value of international trade in ICT goods and services and digitally deliverable services. Based on this, we use export flows of annual statistics on international trade in digitally deliverable services (lnexdigit), which are shown in millions of current United States dollars. When inputting this into the empirical model, we perform logarithmic processing. The data are based on the concept of potentially ICT-enabled services, as developed by UNCTAD in a technical note in 2015, as well as in a report by the 47th United Nations Statistical Commission in 2016.

#### Independent Variables

Abeliansky and Hilbert (2016) measured the impact of data subscriptions per capita and bandwidth per subscription to determine its impact on bilateral exports of goods between countries. Based on this, our paper refers to Abeliansky and Hilbert's research to measure digital infrastructure from the same two perspectives, coverage and convenience, with the former focusing on the extent of access to telecommunication infrastructure that supports trade in digitally deliverable services (Sharma and Gupta, 2003), according to the indicator of fixed broadband subscriptions (per 100 people), while the latter emphasizes measuring the level of development of digital infrastructure, according to the indicator of international bandwidth per Internet user (bit/s).

The fixed broadband subscriptions (per 100 people) (lnpfixed) refer to fixed subscriptions for high-speed access to the public Internet (a TCP/IP connection) at downstream speeds equal to or >256 kbit/s. The data are based on the International Telecommunication Union (ITU) World Telecommunication/ICT Indicators Database and use a weighted average aggregation method. The international bandwidth per Internet user (bit/s) (lnpband) is calculated by converting to bits per second and dividing by the total number of Internet users. The data is based on the International Telecommunication Union (ITU) digital development dashboard.

Hamilton (2000) used current education expenditures to measure investment in human capital, and Vorontsova et al. (2021) used the same indicator as a factor to show the effect on the digitalization gap. Özdogan Özbal (2021) evaluated the long-term effects of high education expenditures and proved that they are positive for the development of human capital through data from OECD countries. Zhylinska et al. (2020) used the number of researchers in R&D (per million people) to characterize the potential to produce human capital. Based on this, this study applies education expenditure (% of GNI) for measurement in terms of educational inputs and adopts researchers in research and development (R&D) (per million people) in terms of educational outputs to reflect a country's quality of education.

Education expenditure (% of GNI) (lneduexgni) refers to the current operating expenditures in education, including wages and salaries, excluding capital investments in buildings and equipment. The data are based on the United Nations Statistics Division's Statistical Yearbook and the UNESCO Institute for Statistics online database estimated by the World Bank, and use a weighted average aggregation method. As for the definition of Research & Development (R&D) (per million people), Researchers are professionals who conduct research and improve or develop concepts, theories, models techniques instrumentation, software of operational methods. Research & Development (R&D) covers basic research, applied research, and experimental development. The data come from the UNESCO Institute for Statistics (uis.unesco.org), and use a weighted average aggregation method.

Pakes and Griliches (1980) examined patents as a measure of innovation indicators by relating them to R&D expenditure. Nurpeisova et al. (2020) use R&D expenditure as a potential innovation variable. According to John (1993), R&D expenditures measure a firm's uniqueness, and Aboody and Lev (2000) see R&D as a source of insider gains. In terms of measurement, Godin (2003) suggests precise definition of what is R&D and which activities fall under or excluded from the heading. Park (2007) studied the theoretical and empirical literature on intellectual property rights (IPRs) to measure innovation related to international technology transfer. Based on these, this study applies research and development expenditure (% of GDP) and charges for the use of intellectual property and payments (BoP, current US$) to measure STI.

Intellectual property payments (BoP, current US$) (lnpintpro) refer to payments between residents and non-residents for the authorized use of proprietary rights (such as patents, trademarks, copyrights, industrial processes and designs including trade secrets, and franchises), and for the use, through licensing agreements, of produced originals or prototypes (such as copyrights on books and manuscripts, computer software, cinematographic works, and sound recordings) and related rights (such as for live performances and television, cable, or satellite broadcast). The data come from the International Monetary Fund, the Balance of Payments Statistics Yearbook, and data files. R&D expenditure (% of GDP) (lnrdpropo) includes both capital and current expenditures in the four main sectors: business enterprise, government, higher education, and private non-profit. R&D also includes basic research, applied research, and experimental development. The data come from the UNESCO Institute for Statistics (uis.unesco.org).

The total population (lnpopu) is based on the de facto definition of the population, which counts all residents, regardless of legal status or citizenship. The values shown are midyear estimates. The data are from the World Bank national account data.

Trade openness has been measured in various ways, the most popular of which is trade share (TS). Referring to Frankel and Romer (1999), Easterly and Levine (2001), and Dollar and Kraay (2004), we use the measure of the ratio of exports plus imports to GDP for that year, in which trade represents the value of all goods and other market services received and provided to the rest of the world. It excludes employees' compensation, investment income (formerly called factor services), and transfer payments. Both trade and GDP data are in current U.S. dollars and come from the World Bank national accounts data and OECD National Accounts data files.

Aizenman and Noy (2006) find linear feedback between trade and foreign direct investment (FDI), which can be accounted for by Granger causality. Chuang and Hsu (2004) argue that the spillover effects of FDI are positive for domestic productivity and thus help companies compete in international markets, using data from China's manufacturing sector. Based on this, we adopt the FDI indicator (lnnetimfdi). This refers to the direct investment equity flows in the reporting economy. Direct investment is a category of cross-border investment associated with a resident in one economy with control or a significant degree of influence on the management of an enterprise resident in another economy. Data are in current U.S. dollars, measure net inflows, and come from the International Monetary Fund, Balance of Payments database, supplemented by data from the United Nations Conference on Trade and Development, and official national sources.

### Regression Model

A multiple linear regression (MLR) model is developed to investigate the factors affecting international trade in digital-global services. In practical use, when interpreting the results of multiple regression, the beta coefficients are valid while holding all other variables constant. The coefficient of determination (R-squared) is used to measure how much of the variation in the outcome can be explained by the variation in the independent variables; the higher the value of R-squared, the better the model fit. For the results of the baseline regression, we also examine how certain “core” regression coefficient estimates behave when the regression specification is modified by adding or removing regressors, which is called the robustness test and was first used by the Ballista project at Carnegie Mellon University. If the coefficients are plausible and robust, they are commonly interpreted as evidence of structural validity. In this study, we use various methods to test robustness: shortening the sample time window and using different indicators to measure digital infrastructure, human capital, and STI.

Model (a) represents the baseline regression and models (b), (c), and (d) replace the indicators measuring digital infrastructure, human capital, and STI, respectively, to test the robustness of the model.


$$\begin{array}{l} lnexdigitx_{it}=\alpha +\beta_{1}lnpband_{it}+\beta_{2}lneduexgni_{it}+\beta_{3}lnpintpro_{it}\\ \;+\;\gamma_{1}lnpopu_{it}+\gamma_{2}lntopen_{it}+\gamma_{3}lnnetimfdi_{it}+\varepsilon_{it}\;(\text{a})\\ lnexdigitx_{it}=\alpha +\beta_{1}lnpfixed_{it}+\beta_{2}lneduexgni_{it}+\beta_{3}lnpintpro_{it}\\ \;+\gamma_{1}lnpopu_{it}+\gamma_{2}lntopen_{it}+\gamma_{3}lnnetimfdi_{it}+\varepsilon_{it}\;(\text{b})\\ lnexdigitx_{it}=\alpha +\beta_{1}lnpband_{it}+\beta_{2}lnrd_{it}+\beta_{3}lnpintpro_{it}\\ \;+\gamma_{1}lnpopu_{it}+\gamma_{2}lntopen_{it}+\gamma_{3}lnnetimfdi_{it}+\varepsilon_{it}\;(\text{c})\\ lnexdigitx_{it}=\alpha +\beta_{1}lnpband_{it}+\beta_{2}lnrd_{it}+\beta_{3}lnrdpropo_{it}\\ \;+\gamma_{1}lnpopu_{it}+\gamma_{2}lntopen_{it}+\gamma_{3}lnnetimfdi_{it}+\varepsilon_{it}\;(\text{d}) \end{array}$$

### Descriptive Statistics

The descriptive statistics of the data are presented in Table 1.

**Table 1:** Descriptive statistics.

**Variables**	**Description**	**Sources**	***N***	**Mean**	**SD**	**Min**.	**Max**.
lnexdigit	Log of export flows on international trade in digitally deliverable services	UNCTAD	512	10.11	1.455	6.601	13.19
lnrd	Log of researchers in R&D (per million people)	UNESCO Institute for Statistics (uis.unesco.org)	405	7.984	0.893	4.494	8.995
lnpintpro	Log of Charges for the use of intellectual property, payments	International Monetary Fund	514	21.92	1.509	17.66	25.30
lnpband	Log of International bandwidth per Internet user (bit/s)	ITU	379	10.72	1.429	6.374	15.94
lnpopu	Log of Total population	World Bank	528	17.18	1.869	12.60	21.07
lnnetimfdi	Log of Foreign direct investment, net inflows	International Monetary Fund	480	23.81	1.482	17.37	27.32
lneduexgni	Log of education expenditure (% of GNI)	World Bank	495	1.518	0.319	0.582	2.123
lntopen	Log of Trade openness	World Bank, OECD National Accounts	528	−0.328	1.917	−5.340	4.864
lnrdpropo	Log of R&D expenditure (% of GDP)	UNESCO Institute for Statistics (uis.unesco.org).	111	−0.300	0.910	−4.053	0.685
lnpfixed	Log of fixed broadband subscriptions (per 100 people)	ITU	379	2.904	1.032	−1.347	3.782

## Results

### VIF Test

Since multiple regression models may often have multicollinearity problems among variables, this study further calculates the variance inflation factors of the variables in the benchmark regression, as shown in Table 2, among which the variance inflation factor of Population (lnpopu) is the largest, standing at 4.21; the variance inflation factor of education expenditure (% of GNI) (lneduexgni) is the smallest, at 1.49; and the average variance inflation factor of the variables is 2.31, which is less than the critical value of 10; therefore, it can be judged that there is no multicollinearity problem among the variables.

**Table 2:** VIF test.

**Variable**	**VIF**	**1/VIF**
lnpopu	4.21	0.237644
lnpintpro	2.71	0.368409
lnpband	1.99	0.501834
lntopen	1.73	0.576527
lnnetimfdi	1.73	0.577236
lneduexgni	1.49	0.672231
Mean VIF	2.31	

### Regression Result

The regressions of model (a) (b) (c) (d) are conducted separately, and the obtained estimation results are shown in Table 3, where column (1) represents the estimation of each coefficient obtained from the full-sample benchmark regression of model (a), columns (2) (3) (4) (5) demonstrate all robustness test analyses. Column (2) is the regression estimation of model (a) by excluding the data from 2005 to 2010, and columns (3), (4), and (5) are the regression estimates of models (b), (c), and (d) by replacing the indicators of digital infrastructure, human capital, and STI capability, respectively.

**Table 3:** Model regression and robust test.

	**(1)**	**(2)**	**(3)**	**(4)**	**(5)**
	**lnexdigit**	**lnexdigit**	**lnexdigit**	**lnexdigit**	**lnexdigit**
	**Full sample year [2015–2020]**	**Year >2010**	**Digital infrastructure replace**	**Human capital replace**	**Technic and innovation replace**
lnpband	0.334***	0.392***		0.339***	0.399***
	(0.0350)	(0.0494)		(0.0353)	(0.0713)
lneduexgni	0.351*	0.418**	0.410**		
	(0.136)	(0.159)	(0.153)		
lnpintpro	0.431***	0.484***	0.544***	0.421***	
	(0.0390)	(0.0469)	(0.0441)	(0.0392)	
lnpopu	0.291***	0.325***	0.0680	0.266***	1.051***
	(0.0415)	(0.0515)	(0.0406)	(0.0408)	(0.0636)
lntopen	0.0500*	0.0594*	0.0125	0.0453	0.119**
	(0.0250)	(0.0290)	(0.0279)	(0.0252)	(0.0420)
lnnetimfdi	0.201***	0.132***	0.225***	0.200***	0.0461
	(0.0303)	(0.0367)	(0.0347)	(0.0306)	(0.0370)
lnpfixed			0.332***		
			(0.0997)		
lnrd				0.511***	0.927***
				(0.0585)	(0.0631)
lnrdpropo					0.159**
					(0.0499)
_cons	−17.18***	−17.85***	−11.47***	−16.10***	−24.17***
	(1.113)	(1.414)	(1.240)	(1.042)	(1.381)
*N*	286	189	286	286	85
r2	0.824	0.841	0.775	0.820	0.935

Standard errors in parentheses *p < 0.05, ^**^p < 0.01, ^***^p < 0.001.

#### Baseline Regression Results

As for the full-sample regression, showed in column (1) in Table 3, our core explanatory variables of particular interest, namely digital infrastructure, human capital, science and technology, and innovation capabilities, have significant positive effects on a country's digitally deliverable services trade competitiveness. Based on the magnitude of the explanatory coefficients, it can be initially determined that science, technology, and innovation capabilities have the strongest effect on a country's digitally deliverable services trade compared to digital infrastructure and human capital. Meanwhile, the population size and trade openness of a country's economy and foreign direct investment also affect a country's digitally deliverable services trade competitiveness. The indicator *R*^2^ which measures the fitting degree of the model, is 0.824, which indicates that the degree of fit is high and that the variables have a strong explanatory power for the model.

#### Robustness Test Regression Results

To further test the robustness of the above findings, showed in columns except (1) in Table 3, the following aspects were analyzed: First, this study selected the years for the regression data, excluding the data of 2010 and before, and ran the same regressions on the subsample, which shows that the core explanatory variables and other explanatory variables maintain a significant positive effect, with the level of STI still playing the most important influence. Second, regarding the different measures of digital infrastructure variables, this study applied fixed broadband subscriptions per 100 inhabitants (lnpfixed) instead of international bandwidth per Internet user (bit/s) (lnpband) in the robustness test for the measurement of digital infrastructure, which demonstrates that the coefficients of the core variables are also significantly positive, and the magnitudes of the coefficients are very similar, supporting the results of the benchmark regression. Third, as for the different measures of human capital variables, this paper adopted researchers in R&D (per million people) (lnrd) to replace education expenditure (% of GNI) (lneduexgni) in the robustness test, which demonstrates that the coefficients of the core variables are significantly positive, and the value of the human capital coefficient shows a more important effect, probably due to the fact that people working in R&D tend to have a higher level of education, work more creatively than in basic jobs, and are more in line with the requirements of the professionals needed for the development of digital trade. Finally, in terms of science, technology, and innovation capabilities, this study used research and development expenditure (% of GDP) (lnrdpropo) instead of charges for the use of intellectual property, payments (BoP, current US$) (lnpintpro), which shows that the coefficients of the core variables are significantly positive, the coefficient value of human capital increases further, and the fitting degree *R*^2^of the whole model is significantly improved.

### Heterogeneity Test Regression Results

Table 4 presents an example. This study carries out a further analysis of the regression results, where high-and middle-income countries in groups (middle-income countries, including Brazil, China, India, Indonesia, Mexico, Russia, South Africa, and Turkey) are categorized for regression. As the results show, the regression results for high-income countries are similar to the full-sample regressions; therefore, the sample data are robust. The regression results for middle-income countries differ on individual non-core explanatory variables due to the limitations of the data sample. Compared with the subsample of high-income countries, the coefficient values of human capital and STI are significantly higher in the subsample of middle-income countries, indicating that trade in digitally deliverable services in middle-income countries is more sensitive to human capital and STI. In particular, the contribution of human capital to the digitally deliverable services trade in middle-income countries is more pronounced. In addition, middle-income countries tend to have a larger population size, which brings more potential for growth in the development of digitally deliverable services. Combined with the level of trade openness, middle-income countries must firmly grasp the strength of trade openness and develop digitally deliverable services in a gradual and progressive manner when aligning with the international community. The foreign direct investment coefficient value is not significant, as digitally deliverable services trade tends to be concentrated in some industries with high technology value. The prospects for foreign direct investment in such countries are limited, which is also in line with the current economic situation in middle-income countries.

**Table 4:** Heterogeneity test.

	**(1)**	**(2)**	**(3)**
	**lnexdigit**	**lnexdigit**	**lnexdigit**
	**Full sample**	**High income countries**	**Middle income countries**
lnpband	0.334***	0.476***	0.0455*
	(0.0350)	(0.0350)	(0.0214)
lneduexgni	0.351*	0.711***	1.095***
	(0.136)	(0.148)	(0.121)
lnpintpro	0.431***	0.392***	0.541***
	(0.0390)	(0.0355)	(0.0503)
lnpopu	0.291***	0.375***	0.831***
	(0.0415)	(0.0371)	(0.0910)
lntopen	0.0500*	0.0737**	0.216***
	(0.0250)	(0.0248)	(0.0393)
lnnetimfdi	0.201***	0.150***	0.0385
	(0.0303)	(0.0285)	(0.0394)
_cons	−17.18***	−15.32***	−21.61***
	(1.113)	(1.179)	(1.042)
*N*	286	215	71
r2	0.824	0.866	0.980

Standard errors in parentheses *p < 0.05, **p < 0.01, ***p < 0.001.

## Discussion

### Development Trend of Digitally Deliverable Services

In this research, we describe the fast growth of digitally deliverable services in international trade and find the factors that can boost its development in different countries. The results can be supported by many scholars, whose researches also show that digitally deliverable services play an increasing growing role around the world (Obashi and Kimura, 2021; Suominen, 2021; Sebayang et al., 2022). Focusing on propositions related to the influencing factors of digital trade and digitally deliverable services, existing papers also found similar factors, such as ICT infrastructure, innovation capacity, and population, etc.

Digital infrastructure is the main influencing factors on digital economy and trade, from our study and existing research. Zatonatska et al. (2019) conducted research on the development of the Internet and e-commerce in European countries, such as Austria, Poland, and Ukraine, and showed that the spread of Internet technology has a significant impact on the development of e-commerce. At the same time, Internet technology and broadband Internet access have spread faster in low-income countries owing to the late diffusion of digital technologies. In contrast, Internet development in high-income countries is relatively mature, and the speed of technology dissemination has slowed. Abeliansky and Hilbert (2016) analyzed the bilateral goods export data of 122 countries by establishing a gravity model and found that the access quantity and quality of telecommunication infrastructure have a significant impact at the same time, and the impact of access quantity on developed countries is more obvious. The impact of access quality on developing countries is more obvious. Tan et al. (2021), by comparing the digital economy of Vietnam and other ASEAN countries, confirmed that ICT infrastructure and ICT such as the Internet are the driving factors of digital economy growth and emphasized the importance of digital government, active participation, and policy orientation.

Innovation capacity and population also show the growth potential and extensive degree of digital service trade. Barun et al. (2019) confirm that the number of people who have access to ICT, thus acquired competencies and skills which boost innovation capacity, plays an important role in the development of the digital economy in the Republic of Belarus. Kovtoniuk et al. (2021) analyzed the main factors influencing the development of digital trade in the United States, the United Kingdom, and China, indicating that the number of network servers and the share of the population using the Internet have a positive effect on the volume of digital trade, while the degree of protection of intellectual property rights has a negative effect. In addition, this study confirms that the largest digital trade growth rate will occur in China, but the most positive economic effects will be realized in the United States. Lu and Fu (2018) studied the factors affecting bilateral digital trade flows between China and other countries and pointed out that the education level of citizens and the availability of scientists and engineers have a significant impact on digital trade, while the digital infrastructure of exporting countries has no effect. They then explained it from the perspective of soft factors such as system and culture. In addition, papers also found that market openness has an effect on digital service trade. González and Ferencz (2018) point out the importance of market openness in the digital transformation of trade. Engaging in service trade, especially in digital services trade, is affected by market access, and from the perspective of policy makers, measures and recommendations for tackling digital trade are provided.

Besides these factors above, this study adopts the idea of multi-factor analysis and focuses on the research object of digitally deliverable service trade. Research confirms that digital infrastructure, human capital, and STI have a significant effect on digitally deliverable services, among them STI has the strongest impact. In addition, the heterogeneity study also provides a theoretical basis for trying to bridge the trade divide in digitally deliverable services. The majority of middle-income countries enjoy their huge market potential contributed by population size, trade environment conditions, etc., in addition to increasing investment in core influencing factors, especially human capital. Therefore, such countries need to give full play to their own advantages in order to consolidate their competitiveness in greater digital trade development and surpass competitors at the right time.

### Limitations and Future Direction

Digitally deliverable trade is an emerging and cross-discipline research field. It is undeniable that this is a significant trend and key area of international trade development in the future. There are a lot of topics worth studying from concept definition, calculation to influencing factors and trade rules. In this paper we studied from the existing databank and available source. Inevitably, there are flaws in comprehensiveness of data and indicators. With the development of statistical methodological, more data will be available for research. And with more detailed bilateral data, we can analyze the network and connection of digital trade among countries. In addition, digital services are also productions of international cooperation, and related to online ordered services. Therefore, with detailed data of different sectors of the digitally service trade, we can analyze the comparative advantage of different countries and value chain of digital service.

## Conclusion

### Main Conclusions

The epidemic has accelerated the development of international trade in digitally deliverable services and its share of international trade in services, the international trade in digitally deliverable services exports grew from about US$1.88 trillion to around US$3.17 trillion from 2008 to 2020, and its share of global services exports grew from 46.30 to 63.55%. This paper carries out a study examines the factors affecting a country's trade in digitally deliverable services, supported by multiple regression models based on panel data of 33 countries' digitally deliverable service trade from 2005 to 2020. The empirical results show that the core variables that affect a country's trade in digitally deliverable services are consistent with the previous hypothesis, with digital infrastructure, human capital, and STI capabilities showing significant effects. It can also be determined that STI has the strongest effect on a country's digitally deliverable services trade compared to digital infrastructure and human capital. Further heterogeneity analysis shows that in terms of scale, developed countries share a large amount of digitally deliverable services trade, while in terms of speed, some developing countries' digitally deliverable services are growing rapidly with potential. Influencing factors also differ between high-and middle-income countries, while middle-income countries are more sensitive to human capital and STI capabilities compared to high income countries. In particular, the contribution of population to the digitally deliverable services trade in middle-income countries is more pronounced.

### Policy Implications

The findings of the current study provide a theoretical basis for the further development of digitally deliverable services trade at the national level and provide a reference for countries to develop targeted national competitive strategies. First, lay out the digital infrastructure construction, give full play to the positive role of the government in the field of digital infrastructure construction; on the one hand, in terms of adoption, implement policy reforms to accelerate the rollout of 4G and 5G, and to further improve network coverage; on the other hand, in terms of scaling, speed up network access velocity, and reduce network tariffs. Second, improving the talent training and introduction system, increasing education investment, and improving the quality of talent, especially outstanding Internet talents, enhance the match between the industries related to digitally deliverable service trade and the talent training system, and tap into the expertise and established teams in the digital content sector. Third, we vigorously developed digital technologies and enhanced innovation capabilities. To drive technological innovation with institutional innovation, build a digital technology innovation system, and systematically improve a country's innovation capacity in digital trade-related industries. In addition, the construction of an innovation incentive model can draw on U.S. practices in the global digital industry to provide property rights incentives and equity protection under market economy conditions to promote the development of digital technology innovation.

Similarly, enterprises can also start from the aforementioned three aspects. Specifically, enterprises can actively adjust their strategies and invest in digitally deliverable services trade-related industries, especially digital infrastructure companies, which may accelerate their migration toward diversified business models. Furthermore, they may actively seek substantial support from development finance institutions to carry out research and development of core technologies as well as key equipment, and continuously integrate high-quality technical resources to achieve technological innovation, technology transfer, and transformation of results. In terms of human capital, mobilize human resources from all aspects; promote the cooperation and integration of industries, universities, and academia; actively carry out digital talent training programs; and create an atmosphere of innovation and motivation to attract high-quality talents with innovative capabilities in the digital era.

## Data Availability Statement

The original contributions presented in the study are included in the article/supplementary material, further inquiries can be directed to the corresponding author/s.

## Author Contributions

All authors listed have made a substantial, direct, and intellectual contribution to the work and approved it for publication.

## Funding

This study was funded by Beijing Social Science Fund, China (Grant No. 21JCC060).

## Conflict of Interest

The authors declare that the research was conducted in the absence of any commercial or financial relationships that could be construed as a potential conflict of interest.

## Publisher's Note

All claims expressed in this article are solely those of the authors and do not necessarily represent those of their affiliated organizations, or those of the publisher, the editors and the reviewers. Any product that may be evaluated in this article, or claim that may be made by its manufacturer, is not guaranteed or endorsed by the publisher.
